# Conservative Treatment of Spontaneous Spinal Epidural Hematoma: A Report of Two Cases With Severe Neurologic Deficits and a Literature Review

**DOI:** 10.7759/cureus.106360

**Published:** 2026-04-03

**Authors:** Stephanie Wuyts, Joost Dejaegher

**Affiliations:** 1 Neurosurgery, Catholic University of Leuven (KU Leuven), University Hospitals Leuven (UZ Leuven), Leuven, BEL

**Keywords:** acute spinal cord compression, conservative treatment, neurologic recovery, severe neurologic deficits, spontaneous spinal epidural hematoma (sseh)

## Abstract

Spontaneous spinal epidural hematoma (SSEH) is a rare but potentially devastating cause of acute spinal cord compression. Urgent surgical decompression is generally recommended, particularly in patients with severe neurological deficits. However, an increasing number of reports describe conservative management in patients with mild neurological deficits or spontaneous recovery.

We present two cases of cervical SSEH that presented with severe neurological deficits (American Spinal Injury Association (ASIA) B) and were managed conservatively after early spontaneous neurological improvement. In addition, a review of the literature was performed, focusing on prognostic factors and the role of conservative treatment.

Both patients experienced rapid neurological improvement shortly after presentation and achieved complete functional recovery without surgical intervention. Follow-up MRI confirmed complete resolution of the epidural hematoma without evidence of spinal cord injury or underlying pathology. Literature review suggests that patients with mild neurologic deficits or early neurological improvement are candidates for conservative treatment.

While urgent surgical decompression remains the standard of care for SSEH with severe or progressive neurological deficits, conservative management may be a safe and effective alternative in patients demonstrating early spontaneous neurological recovery, even in cases with initial severe neurological deficits.

## Introduction

Spontaneous spinal epidural hematoma (SSEH) is an uncommon but potentially devastating clinical entity. SSEH is characterized by the accumulation of blood within the epidural space of the spinal canal in the absence of trauma or iatrogenic causes, such as spinal surgery or lumbar puncture [1,2]. The estimated annual incidence is approximately 0.1 per 100,000 individuals, accounting for less than 1% of spinal canal-occupying lesions, making it a rare cause of acute spinal cord compression [3,4].

SSEH can affect patients across all age groups, with a reported median age between 50 and 60 years and a slight male predominance [1,2,5,6]. Proposed risk factors include antithrombotic therapy, coagulopathies, hypertension, vascular malformations, and sudden increases in venous pressure due to exertion or Valsalva maneuvers. Nevertheless, in a considerable proportion of cases, no underlying etiology can be identified [1,2,4-6].

Clinically, SSEH presents with a sudden onset of severe spinal or radicular pain followed by rapidly evolving neurological deficits with varying degrees of motor, sensory, and sphincter dysfunction [5,6]. In the acute setting, computed tomography (CT) is often performed when suspecting a SSEH, showing a hyperdense lesion. However, magnetic resonance imaging (MRI) is the diagnostic modality of choice, as it confirms the diagnosis, assesses the extent of the hematoma, and excludes other or underlying pathologies, such as disc herniation, infection, neoplasm, or vascular malformation [7].

SSEH is considered a neurosurgical emergency, with the degree of neurological deficit serving as the principal factor guiding management [8,9]. Urgent surgical decompression via (hemi-)laminectomy with hematoma evacuation is regarded as the gold standard of treatment, aiming to relieve spinal cord compression and prevent irreversible spinal cord injury [10,11]. However, an increasing number of case reports and case series have described patients with mild neurologic deficits or early spontaneous neurologic recovery who were successfully managed conservatively, achieving favorable outcomes without surgical intervention [8,12-15].

In this article, we present two cases of SSEH with severe neurological deficits at presentation, both of which demonstrated early spontaneous improvement and were managed conservatively. In addition, we provide a review of the current literature on SSEH, with a particular emphasis on conservative treatment strategies and cases demonstrating spontaneous neurological recovery. Pubmed was used as a database with "spontaneous spinal epidural hematoma AND conservative OR spontaneous recovery" as search terms; references were screened for additional relevant articles.

Through these cases and the literature review, we aim to contribute to the ongoing discussion on individualized management approaches for this rare but clinically significant condition.

## Case presentation

Both cases were treated in the University Hospital of Leuven in the summer of 2025.

Case 1

A 74-year-old woman was transferred to a nearby regional hospital after contacting emergency services for the sudden onset of severe interscapular pain radiating to all four extremities. Her medical history was notable for hypertension and chronic low-dose aspirin therapy for secondary prevention following reversible myocardial ischemia documented on a myocardial perfusion imaging with technetium-99m methoxyisobutylisonitrile (MIBI) scan. Upon arrival at the emergency department, her condition deteriorated, with progressive sensorimotor deficits developing in the lower extremities. There was no history of recent trauma. On examination by the neurologist at the emergency department, she exhibited complete paraplegia with a T4 sensory level and areflexia in both lower limbs. Sacral sparing was not evaluated, which prevents distinction between American Spinal Injury Association (ASIA) Impairment Scale grades A and B [16]. Cranial nerve function and upper-extremity sensorimotor examination were normal.

**Figure 1:**
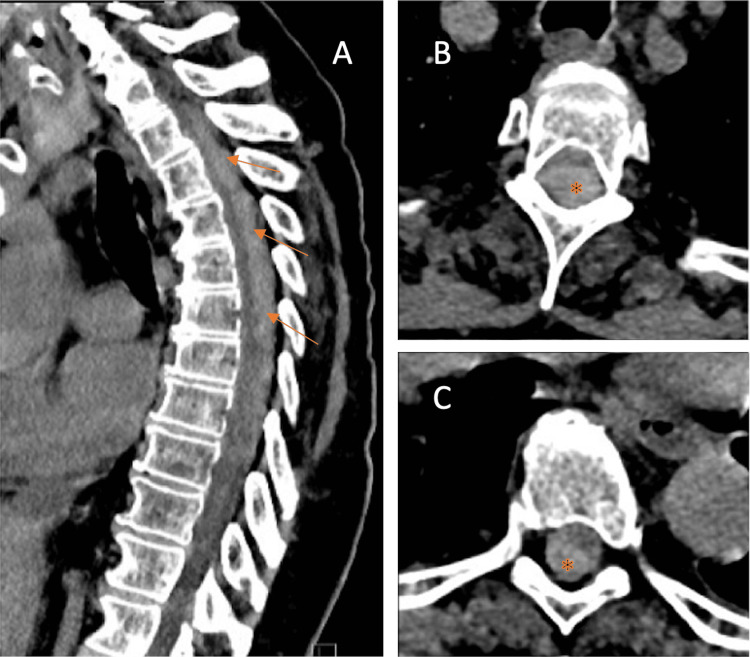
Computed tomography (CT) of the thoracic spine in the first case at presentation, demonstrating an epidural hematoma (arrows and asterisks) extending from T1 to T9. (A) Sagittal image; (B) axial image at T3; (C) axial image at T7.

Non-contrast cranial CT showed no abnormalities. CT of the thorax and abdomen ruled out an aortic dissection or aneurysm. Thoracolumbar CT (Figure 1) revealed a 1 cm-thick hyperdense dorsal epidural mass extending from approximately T1-T2 to T9, suggestive of an epidural hematoma with spinal cord compression.

**Figure 2:**
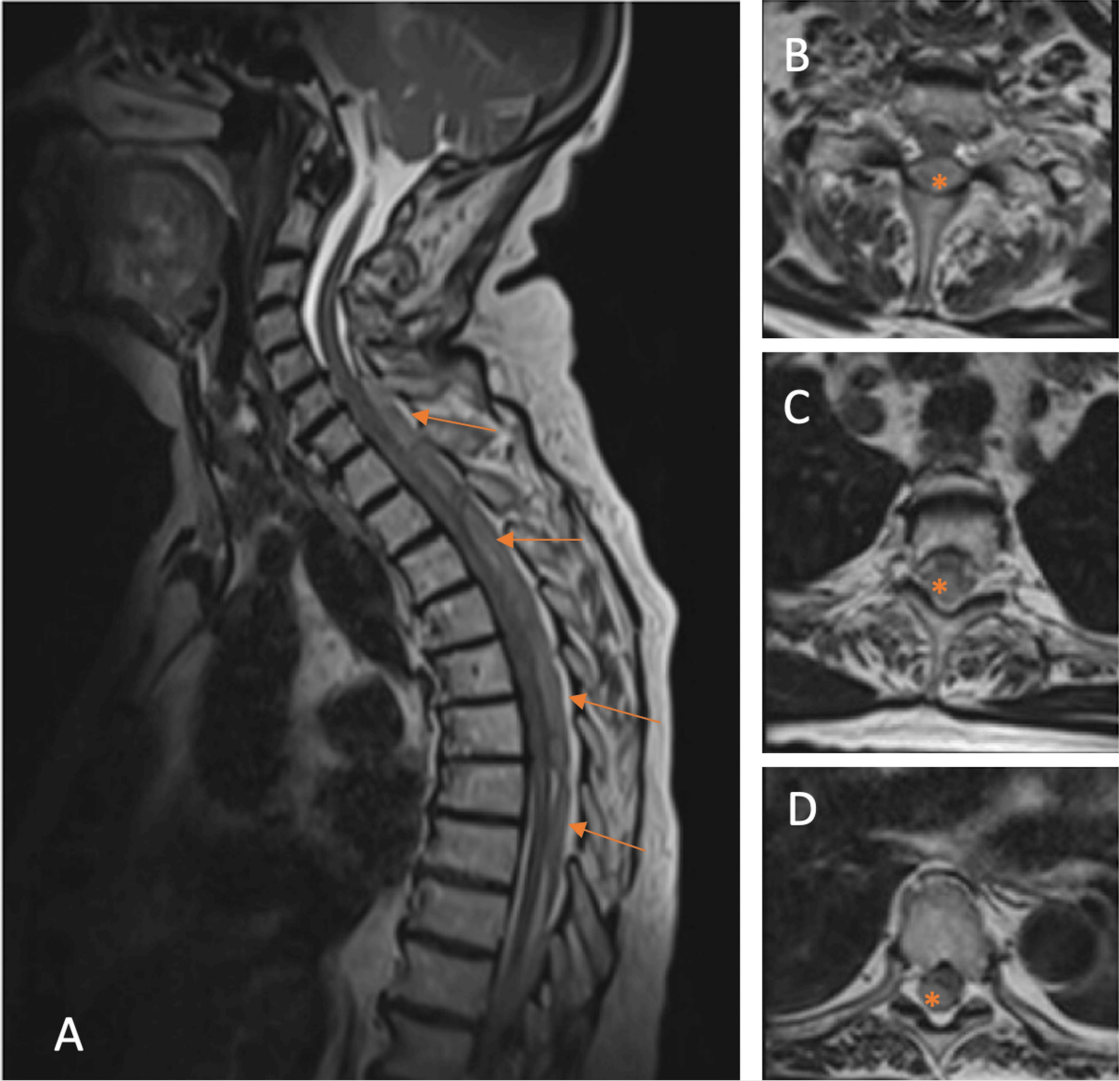
Magnetic resonance imaging (MRI) of the first case at presentation, demonstrating an epidural spinal hematoma (arrows and asterisks) extending from C5 to T8. (A) Sagittal T2-weighted image; (B) axial T2-weighted image at C7; (C) axial T2-weighted image at T3; (D) axial T2-weighted image at T6.

The patient was subsequently transferred to our tertiary care center. Upon arrival, her neurological status had markedly improved: motor function was fully restored with normal reflexes and only mild residual sensory disturbances in the lower extremities. Urgent MRI (Figure 2) of the entire spine confirmed a dorsally located spinal epidural hematoma extending from C5 to T8, causing significant compression of the spinal cord but without medullary signal alterations. Laboratory results, including coagulation studies, were normal.

The paraplegia had lasted approximately one hour and gradually resolved to complete neurological recovery prior to arrival. Given the rapid and substantial spontaneous improvement, a conservative approach was adopted.

The patient was admitted to the intensive care unit for close neurological monitoring, including hourly examination by a neurosurgery resident, and maintenance of mean arterial pressure (MAP) above 80 mmHg using a noradrenaline infusion in analogy to the protocol in spinal cord injury [17]. Aspirin was discontinued and, following consultation with the hematology and vascular medicine teams, was permanently withheld. After three days, she was transferred to the neurosurgical ward, and she was discharged home on day 7 without any neurological sequelae. No additional bleeding diathesis or other precipitating factor was identified apart from aspirin use.

**Figure 3:**
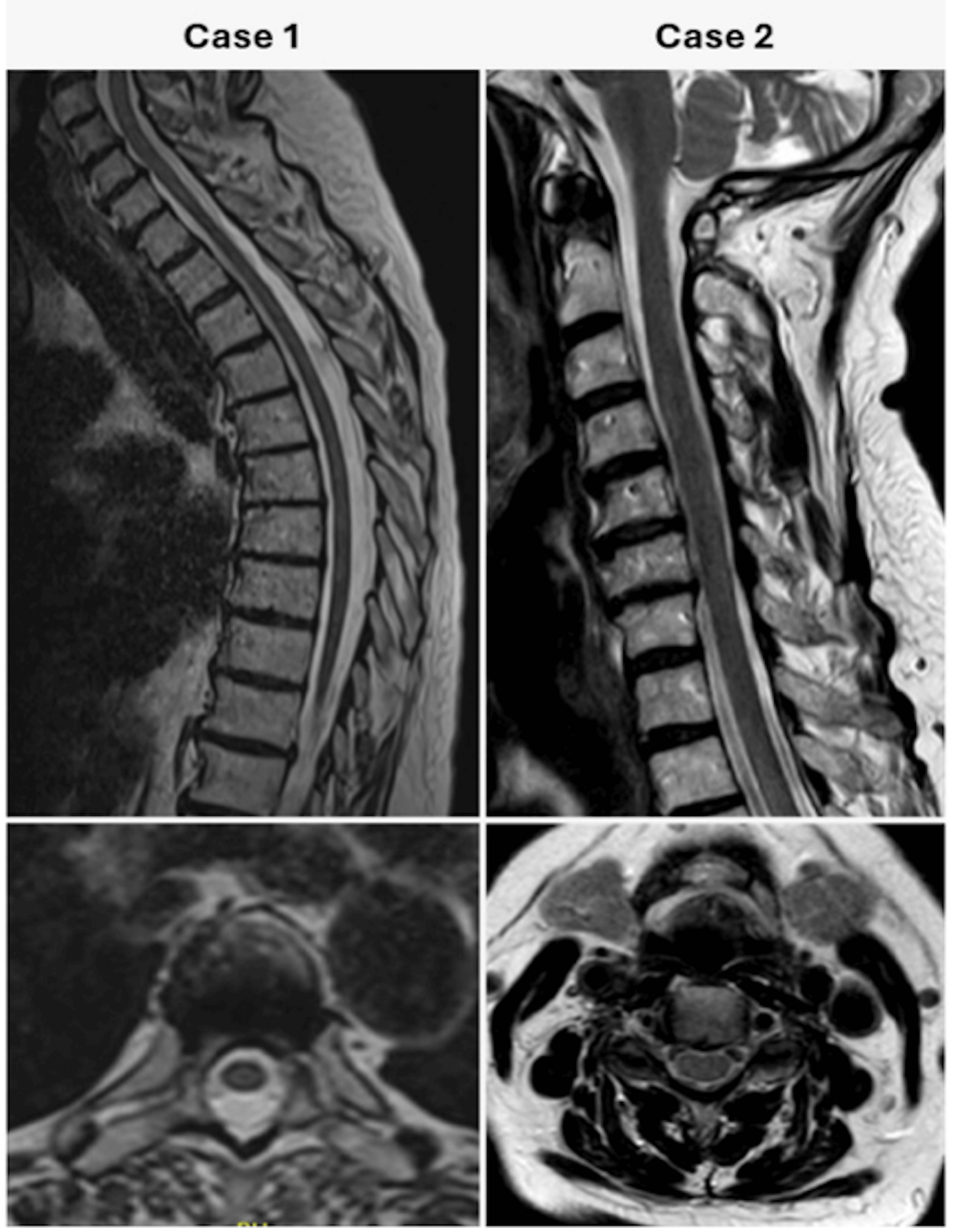
Sagittal (upper) and axial (lower) T2-weighted magnetic resonance imaging (MRI) at follow-up, demonstrating no residual hematoma, spinal cord injury, or underlying lesion.

At the six-week follow-up, the patient remained asymptomatic, with a normal neurological examination. Follow-up MRI (Figure 3) demonstrated complete resolution of the epidural hematoma, with no evidence of myelomalacia or an underlying lesion such as a vascular malformation or tumor.

Case 2

A 74-year-old woman awoke with severe, stabbing cervical pain radiating to both shoulders, predominantly the right. Shortly thereafter, she developed a right-sided sensorimotor hemiparesis, prompting emergency medical services to be contacted. Her medical history included hypertension and a transient ischemic attack 18 years earlier, for which she was taking low-dose aspirin.

**Figure 4:**
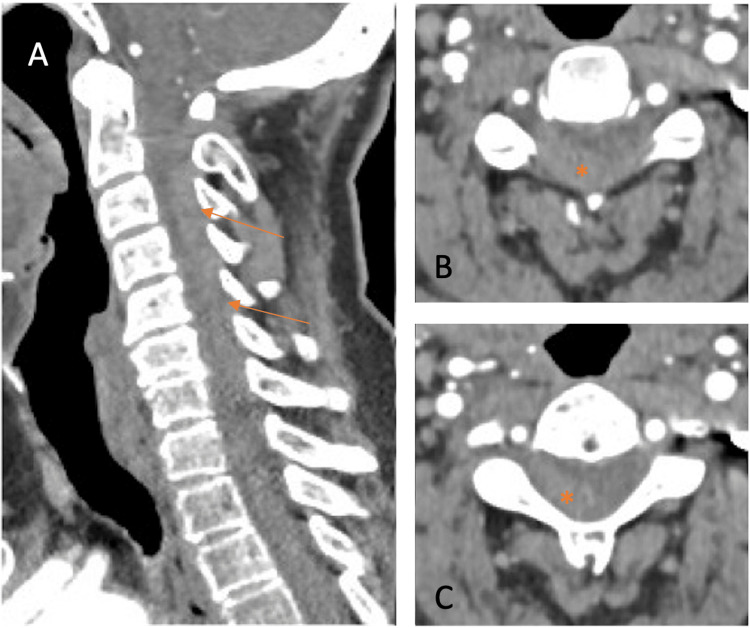
Computed tomography (CT) angiogram of the second case suggesting a cervical spontaneous spinal epidural hematoma (SSEH) (arrows and asterisks) on the right posterolateral side extending from C2 to C7. (A) Sagittal image; (B) axial image at C3; (C) axial image at C5.

Neurological examination at the emergency department revealed right-sided hemiplegia without facial involvement. Non-contrast head CT was unremarkable, showing no early ischemic changes or intracranial hemorrhage. CT angiography (Figure 4) revealed a suspected right-sided dorsal epidural collection with an approximate thickness of 8 mm extending from C2 to C7. Following imaging, her neurological condition deteriorated further, with new-onset motor deficits on the left side.

Neurosurgical assessment demonstrated complete loss of motor function in both lower extremities and the right upper extremity, and mild weakness of the left upper extremity (Medical Research Council (MRC) grade 4/5). Cranial nerve function was intact, deep tendon reflexes were absent, and both Babinski and Hoffman-Trömner signs were negative. Sensory examination was normal, concluding to an ASIA B. Immediately after this assessment, a spontaneous improvement of left-sided motor function was observed, with recovery to MRC 4/5 in the left leg and improved movement in the left arm, while a dense right-sided hemiplegia persisted. Laboratory results, including coagulation studies, were normal.

**Figure 5:**
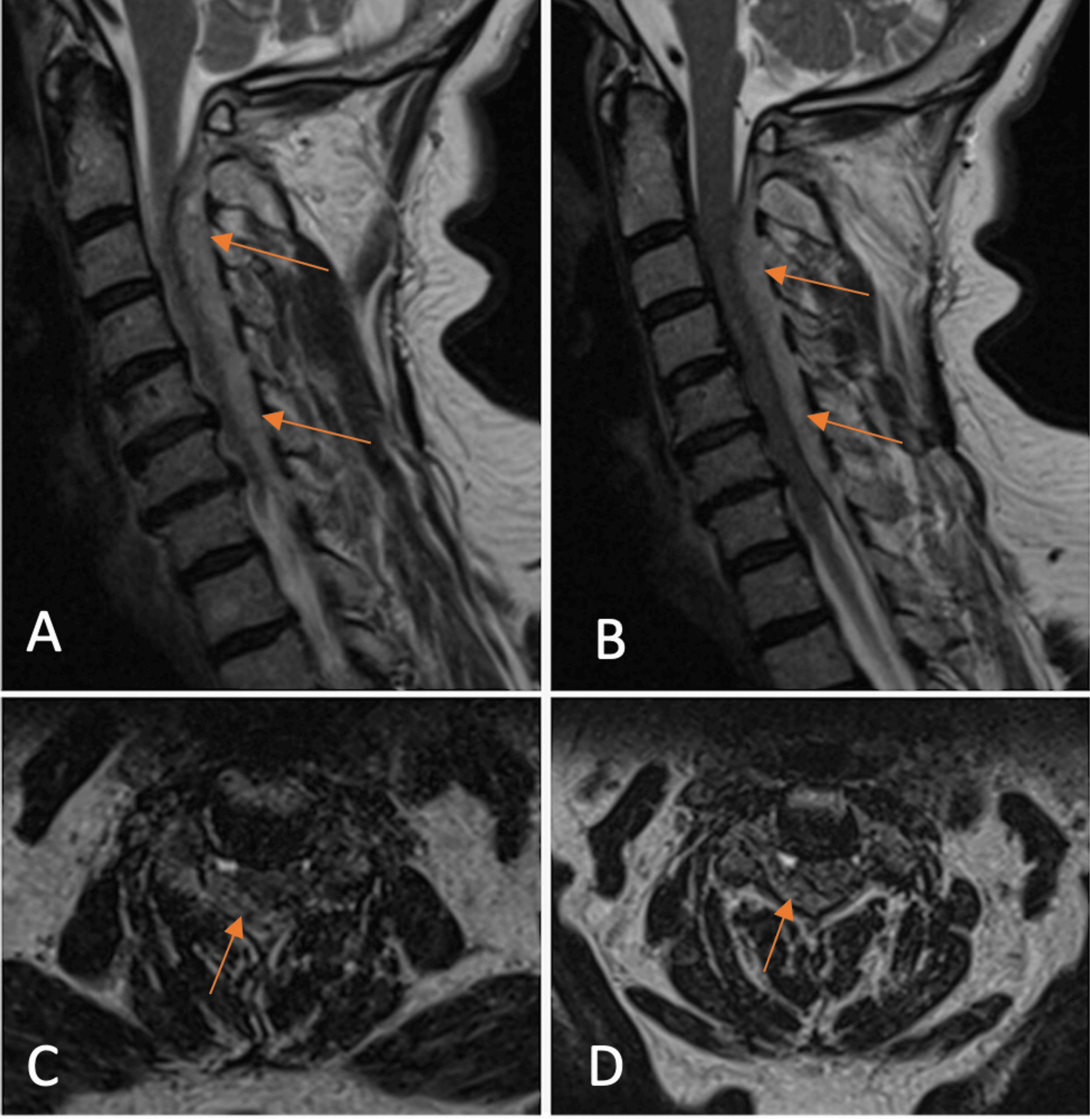
T2-weighted magnetic resonance imaging (MRI) of the second case demonstrating a spontaneous spinal epidural hematoma (SSEH) (arrows) on the right posterolateral side extending from C2 to C7. (A) Sagittal image paramedian on the right side; (B) midline sagittal image; (C) axial image at C3; (D) axial image at C5.

Urgent MRI of the entire cervical and upper thoracic spine (Figure 5) demonstrated a homogeneous dorsal epidural collection - T2-hyperintense and T1-hypointense - predominantly on the right side and extending from C2 to T1, consistent with a spinal epidural hematoma causing significant cord compression. After imaging, additional improvement in right-sided motor function was observed (MRC 3/5).

Due to the ongoing spontaneous improvement, a conservative approach was adopted; however, a low threshold for surgery was maintained depending on the evolution. The patient was admitted to the high-care area of the emergency department for hourly neurological monitoring, where continuing neurologic improvement was documented (MRC 4/5 and partially 5/5). She was subsequently transferred to the intensive care unit for close neurological observation and blood pressure augmentation, targeting a MAP above 80 mmHg using low-dose noradrenaline. Aspirin was withheld and reversed with two units of platelets and desmopressin. Upon ICU admission, approximately five hours after initial presentation, motor function was nearly normal, with only mild residual weakness (MRC 4+/5) in right shoulder abduction and elbow flexion.

After five days, the patient was transferred to the neurosurgical ward. At that time, clinical examination showed persistent mild weakness in right shoulder abduction (MRC 4+/5) with subtle arm drift during the Barré maneuver. She also continued to report right-sided shoulder pain. At hospital discharge four days later, the right shoulder abduction remained MRC 4+/5 but without arm drift, showing only minimal pronation during the Barré test, and no functional limitations. The patient reported mild paresthesia in the sole of the right foot and posterior part of the lower leg, as well as residual right-sided shoulder pain. Reflexes were normal.

At four-week follow-up, the patient reported persistent right shoulder discomfort and neuropathic pain in the posterior aspect of the right lower leg and foot, for which pregabalin was prescribed. Neurological examination revealed no remaining motor deficits. Repeat spinal MRI (Figure 3) showed that the epidural hematoma had fully resolved, and there were no imaging features suggestive of spinal cord injury or an underlying causative abnormality. At three months follow-up, the patient no longer experienced bothersome symptoms. Given the spontaneous improvement, the patient did not initiate the proposed treatment with pregabalin.

## Discussion

SSEH is a rare clinical entity, with a commonly cited incidence of 0.1 per 100,000 individuals per year [3]. However, this estimate is based on data that are several decades old. Given the widespread availability and routine use of MRI, it is plausible that the incidence has increased as the condition is now more frequently recognized [8]. This assumption is further supported by discrepancies in reported hematoma localization. While Holtas et al. described ventral hematomas twice as frequently as dorsal ones, the majority of subsequent literature reports a predominantly dorsal or posterolateral location, with posterior involvement described in more than 75-90% of cases [1,2,5,18]. It is conceivable that smaller or less symptomatic SSEHs, particularly those presenting with pain alone, remain underdiagnosed, as urgent imaging is only indicated in patients with neurological impairment.

Epidemiologically, SSEH demonstrates a bimodal age distribution, with peak incidence in the second and sixth-seventh decade of life, and a slight male predominance of approximately 60% [1,5]. The location of SSEHs follows a similar distribution; cervical and cervicothoracic locations are most common across all age groups, whereas thoracolumbar involvement increases with advancing age. On average, SSEH extend across three to four vertebral segments [1,2,5].

The etiology of SSEH remains debated; both venous and arterial sources have been proposed, as well as underlying vascular malformations [2,19,20]. A venous origin is most commonly postulated, involving rupture of the internal vertebral venous plexus of Batson. The valveless nature of this plexus renders it susceptible to abrupt increases in thoracic or abdominal pressure, potentially leading to rupture at a locus minoris resistentiae [2,19]. In contrast, Beatty and Winston proposed an arterial source, arguing that the rapid onset of symptoms and the relatively low venous pressure compared to intrathecal pressure in the cervical spine favor arterial bleeding from an anastomotic vessel connected to a radicular artery, triggered by trauma or abrupt movement [21]. Groen and Ponssen countered this hypothesis by demonstrating a prominent posterior venous plexus at the C5-T2 level - the most common location of SSEH - which becomes increasingly developed with age, particularly in the thoracolumbar region [22]. These anatomical observations align with epidemiological findings and support a venous etiology as the most plausible explanation. However, the clinical relevance of determining the precise bleeding source remains limited at this time.

Clinically, SSEH typically presents with sudden onset neck or back pain, sometimes radiating to the extremities, followed by neurological deficits including motor weakness, sensory disturbances, and occasionally sphincter dysfunction. The severity of deficits ranges from mild paresis to complete paralysis and is commonly graded using the ASIA Impairment Scale or Frankel score [2,5,6]. Although paraparesis or quadriparesis is most frequently observed, Brown-Séquard syndrome and hemiparesis have also been described [23-26]. The latter may mimic acute ischemic stroke [23]. Pain, present in approximately 80-90% of SSEH cases, together with the absence of cranial nerve involvement or cortical signs, can aid in the differentiation [1,2,23]​​​​​​. Nonetheless, caution is warranted, as conditions such as carotid artery dissection can also present with painful hemiparesis [23]​​​​​​. Inatomi et al. reported that 0.2% of suspected stroke patients were ultimately diagnosed as SSEH; notably, 80% of these patients experienced spontaneous neurological recovery, and only one required surgical decompression [27]​​​​​​. Importantly, CT angiography, routinely performed in acute stroke work-up, may reveal findings suggestive of an SSEH and should prompt further spinal imaging [23]​​​​​​. Accurate differentiation is crucial, as administration of thrombolytic therapy in SSEH has been associated with neurological deterioration [23]​​​​​​.

The role of antiplatelet and anticoagulant therapy in the pathogenesis and management of SSEH remains controversial. While several case reports implicate antiplatelet agents such as acetylsalicylic acid (ASA) and clopidogrel as potential precipitating factors [19]​​​​​​, a large meta-analysis found no increased prevalence of antiplatelet therapy use among SSEH patients compared with the general population, arguing against ASA as an independent risk factor [2]​​​​​​. In contrast, vitamin K antagonists such as warfarin have consistently been associated with both an increased risk of SSEH and poorer neurological outcomes [2,5,6]​​​​​​. Patients not on warfarin were 2.9 times more likely to have a better postoperative outcome [6]​​​​​​. Evidence regarding direct oral anticoagulants (DOACs) remains limited to case reports and a single case series, thus providing insufficient evidence to draw firm conclusions [28-30]​​​​​​.

Management of SSEH in the context of coagulopathy is equally debated. Some authors advocate immediate correction of coagulation abnormalities to prevent hematoma progression and facilitate neurological stabilization [31]​​​​​​. Others have proposed that impaired coagulation may prolong the liquefied state of the hematoma, facilitating its longitudinal spread within the epidural space and thereby reducing focal spinal cord compression [8,12,32]​​​​​​. This hypothesis may partly explain why some antithrombotic-associated SSEHs, including the cases presented here, follow a relatively benign course under conservative management. However, this feature is only relevant for the blood in the epidural space, not the normal endovascular blood, where normal clotting is preferred to prevent further bleeding. Overall, reversal of antithrombotic medication, particularly vitamin K antagonists, is generally advocated, given their association with worse prognosis and the potential need for urgent surgical intervention [31]​​​​​​.

Surgical decompression via laminectomy or hemilaminectomy with hematoma evacuation remains the standard treatment for SSEH, particularly in patients with severe neurological deficits (ASIA A-B) or clinical deterioration [5,8]. Conservative management is generally considered appropriate in patients with mild deficits (ASIA D-E) or early spontaneous neurological recovery [9,13,31]​​​​​​. Several authors have extended this approach to selected ASIA C patients demonstrating early improvement [14,33]​​​​​​. Fukui et al. demonstrated comparable outcomes between surgically and conservatively treated ASIA C patients across different centers in Japan [13]​​​​​​. Notably, the present cases expand this paradigm by demonstrating favorable outcomes in patients who initially presented with severe deficits (ASIA B) but exhibited rapid spontaneous neurological improvement. While the literature predominantly supports conservative treatment in milder cases, these observations suggest that early dynamic neurological improvement may be more relevant than initial ASIA grade alone.

Although conservative management is frequently mentioned in the literature as an option for patients exhibiting early spontaneous neurological improvement, specific guidance regarding the timeframe within which improvement should occur, or the degree of recovery that qualifies as “sufficient”, is lacking [33]​​​​​​. In a review by Zhang et al., 79% of conservatively managed patients show complete recovery, with 63% showing improvement within the first 24 hours [15]. This consideration is particularly important, as patient outcome is closely linked to the timing of surgical intervention. Optimal timing, however, remains debated: some studies recommend surgery within 24 hours of symptom onset [2,13]​​​​​​, while others advocate intervention within 12 hours to maximize neurological recovery [6,34,35]​​​​​​. Nakamura et al. demonstrated 100% of patients operated on within 12 hours of onset achieved good outcomes (ASIA D or E), compared with 63.6% of those operated on after 12 hours. Additionally, patients whose ASIA grade improved by ≥2 grades had a significantly shorter median time to surgery than those improving by ≤1 grade (8 vs. 14 hours) [34]. The authors noted, however, that some of the observed improvement could have occurred with conservative treatment, potentially exaggerating the apparent effect of surgery [34]​​​​​​.

Overall in the literature, 23-33% of patients are managed conservatively, with 73-84% achieving complete neurological recovery (ASIA E) [5,9]​​​​​​. In contrast, patients undergoing surgery - generally those with more severe neurological deficits - demonstrate complete recovery in 37-48% of cases [2,9]​​​​​​. The key literature on conservative treatment is summarized in Table 1.

**Table 1:** Summary of key literature on conservative treatment ASIA: American Spinal Injury Association Impairment Scale; MRI: magnetic resonance imaging

Study (year, country)	Design/period	Patients (n)	Initial neurological status	Treatment groups	Key outcomes	Main conclusion
Zhang et al. [15] 2019, China	Literature review + 2 own cases (1989-2019)	19	ASIA A: 5; ASIA B-D: 6; ASIA E: 3; unknown: 5	All conservative: 11/19 spontaneous improvement, 2 patient refusal, 2 mild symptoms, 2 delayed or misdiagnosis	63.2% neurological improvement within 24 h; 79% complete recovery and 74% complete MRI resolution within one month	Conservative management is appropriate in mild deficits or early spontaneous recovery with close monitoring; surgery if no improvement within 24h in severe cases or if deterioration occurs
Wang et al. [36] 2017, China	Retrospective case series (2010-2016)	24	Surgery: ASIA A-C (19); Conservative: ASIA D-E (5)	Decompressive surgery vs conservative treatment	Surgery: 37% good outcome (ASIA A/B to ASIA C or ASIA C to ASIA D). Conservative: 100% good outcome (ASIA E), MRI resorption after 2 weeks	Conservative treatment is suitable for ASIA D-E; surgery is recommended for ASIA A-C
Kim et al. [14] 2012, Korea	Retrospective case series (2004-2012)	15: 10 surgery 5 conservative	Surgery: ASIA A-D; Conservative: ASIA C-E	Surgery vs conservative (dexamethasone)	Outcome strongly correlated with initial ASIA grade; no significant difference between treatments	Early surgery advised; conservative management acceptable in ASIA E or ASIA C-D with early recovery
Nitta et al. [12] 2025, Japan	Retrospective single-center observational study (2009-2023)	44 total: 29 conservative, 7 excluded: 22 (4 no MRI, 2 delayed diagnosis, 1 delayed surgery)	Conservative group mainly ASIA D-E (59% ASIA E)	Conservative management (1 delayed surgery excluded)	MRI after five days showed hematoma size reduction in all patients (no complete resolution)	Conservative treatment is feasible in improving or minimal deficits with close follow-up
Fukui et al. [13] 2022, Japan	Retrospective multicenter case-control (2009-2019)	62	Frankel A-B only surgical; D-E only conservative; C both	Surgery vs conservative	In all patients: surgery, older patients, more thoracic location, worse Frankel grade before and after treatment. In Frankel C, no difference in any parameters between surgery and conservative, except for facilities	Frankel C: prognosis non-inferior with conservative treatment: Conservative treatment is a valid option in Frankel C patients
Raasck et al. [9] 2017, Canada	Review + case series (2000-2015)	65 (50 surgery, 15 conservative) + 6 (5 surgery, 1 conservative)	Surgery: 64% ASIA A-B, 20% D-E. Conservative: 33% ASIA A-B, 53% ASIA D (Significant difference in pre-treatment neurologic deficit; however, still 33% ASIA A-B in conservative management)	Surgery vs conservative	Post-op: Surgery: 18% ASIA A-B, 26% ASIA D, 48% ASIA E Conservative: 27% ASIA D, 73% ASIA E, 73% full recovery in the conservative group vs 48% in the surgery group; all ASIA C-D improved, 30% ASIA A didn’t	Conservative management is effective when spontaneous recovery is present
Groen [8] 2004, The Netherlands	Review (1988-2004)	64 conservative vs 474 surgical	Conservative cases had milder deficits	Conservative vs surgery	84% complete recovery in the conservative group; longer hematomas (5.4 vs 4.2 levels). More diagnosis based on MRI	Surgery remains standard; conservative treatment is only for mild deficits or early improvement with strict monitoring

The most robust predictor of outcome remains the severity of neurological impairment at presentation. Incomplete deficits are consistently associated with better recovery than complete deficits [6,35,37]​​​​​​. A review by Mukerji and Todd reported full neurologic recovery in 56% of patients with incomplete spinal cord injury compared to 27% in those with complete spinal cord injury [35]​​​​​​. Bakker et al. further noted that even partial preservation of sensory function in the context of a complete motor deficit increased the likelihood of a favorable outcome by a factor of 4.6 [2]​​​​​​. A more recent review from 2023 reported ASIA B, C, and D were 3.71, 5.75, and 24.99 times more likely, respectively, to achieve better post-operative outcomes compared to ASIA A patients [6]​​​​​​.

Hematoma characteristics further impact prognosis. A thoracic location is the only independent predictor of more severe pre-treatment neurological deficits [13]. Thoracic SSEH is also associated with poorer outcomes, likely due to the narrower spinal canal and relatively vulnerable vascular supply of the thoracic cord [2,5]​​​​​​. Several studies associate hematoma extension over more than four vertebral segments with worse neurological outcomes [2,6,31]​​​​​​. Paradoxically, Groen observed that conservatively treated patients had a greater mean hematoma length (5.4 vs. 4.2 vertebral levels), which may reflect longitudinal redistribution [8]​​​​​​. An additional parameter to consider is the percentage of spinal canal obliteration by the hematoma as described by Marhold et al. [38]. Demonstrating a lower mean obliteration of 46.4% in patients with a favorable outcome compared to 62.1% in those with an unfavorable outcome, proposing a threshold of 51% as a prognostic cut-off [38]. A recent case series of 47 patients reported a trend toward longer hematoma extension (median 6 vs. 3 vertebral levels) and higher canal occupancy ratios (median 63.6% vs. 57.1%) in patients with poor outcomes, although these differences did not reach statistical significance [18]​​​​​​. Katayama et al. did​​​​​, however,​​​​identify perioperative hypotension as a significant predictor of poor neurological outcome [18]​​​​​​. Blood pressure management is therefore critical, particularly during anesthesia and postoperative care. Maintaining spinal cord perfusion with normotensive targets and MAP augmentation is recommended, avoiding hypotension that may exacerbate ischemia and excessive hypertension that may promote ongoing hemorrhage [18,36]​​​​​​. Hypertension itself does not appear to be associated with worse outcomes [2,5]​​​​​​. Additional possible adverse prognostic factors are increased age [2]​​​​​​, rapid neurological deterioration [2,5]​​​​​​, sphincter disturbances [5]​​​​​​, and the presence of myelomalacia on MRI [38,39]​​​​​​. Endo et al. proposed reduced apparent diffusion coefficient (ADC) values in T2 hyperintense lesions of the spinal cord as an indicator of ischemia and irreversible spinal cord damage, highlighting the potential role of advanced MRI parameters in prognostic stratification [40]​​​​​​.

The natural history of SSEH remains incompletely understood. Increasing reports of spontaneous neurological recovery have led to several hypotheses regarding underlying mechanisms. The observation that conservatively treated SSEHs tend to have a greater longitudinal extent may indicate redistribution of the hematoma along the spinal epidural space, resulting in reduced transverse spinal canal obliteration and thus decreased focal mass effect on the spinal cord [8]​​​​​​. This finding supports the hypothesis that early neurological improvement may, at least in part, be driven by longitudinal hematoma redistribution rather than by rapid resorption. In support of this hypothesis, several studies have reported neurological improvement preceding radiological resolution of the hematoma [41]​​​​​​. However, this interpretation is not universally accepted. Nagata et al. proposed that hematoma resolution occurs predominantly in situ, based on follow-up MRI demonstrating both a reduction in hematoma size without longitudinal spread and an increase in spinal cord cross-sectional area after 7-16 days, findings they attributed to clot liquefaction and subsequent decompression rather than longitudinal redistribution [41]​​​​​​. It is possible, however, that early imaging may not capture dynamic changes occurring shortly after symptom onset, and it cannot be excluded that partial redistribution had already taken place before the initial MRI in patients with early neurological recovery. An alternative, non-mutually exclusive explanation for early neurological improvement is an initial phase of "spinal shock" induced by the abrupt mass effect on the spinal cord, followed by partial neurological recovery as the cord adapts to the compressive insult. Experimental work by Tarlov et al. demonstrated that the development and reversibility of neurological deficits depend on the magnitude of compression, its duration, and how rapidly the compressive force was applied [42-44]​​​​​​. These hypotheses lack strong evidence, but a better understanding of the pathophysiology may improve clinical decision-making.

The two cases presented in this article are notable for early spontaneous neurological improvement despite initially severe deficits (ASIA B). While the current literature predominantly supports conservative management in patients with mild neurological deficits (ASIA D-E) and some evidence towards non-inferiority of conservative management in ASIA C patients [13]​​​​​​, these cases suggest that favorable recovery may also occur in patients with more severe initial deficits when early neurological improvement is observed. Both patients were managed with intensive neurological monitoring, hemodynamic monitoring with normotensive blood pressure targets MAP >80 mmHg to optimize spinal cord perfusion, and a low threshold for immediate surgical intervention in the event of neurological deterioration. Importantly, this observation should not be interpreted as an argument to delay surgery in patients with severe SSEH. Rather, it highlights the need for individualized decision-making based on dynamic clinical assessment rather than reliance on initial neurological classification alone.

Limitations

As SSEH remains a rare condition, the existing literature on SSEH is almost exclusively retrospective and subject to substantial selection bias, as patients with severe deficits are more likely to undergo surgery, whereas conservatively managed cohorts tend to include milder cases. Consequently, direct comparison between treatment strategies remains challenging, and high-quality prospective data are lacking.

## Conclusions

SSEH is a rare but potentially devastating cause of acute spinal cord compression that requires rapid recognition and careful clinical decision-making. While urgent surgical decompression remains the treatment of choice for most patients with SSEH, conservative management may be safely considered in a carefully selected subgroup demonstrating mild neurologic deficits or early and sustained neurological improvement, even in the presence of initially severe deficits. Strict neurological and hemodynamic monitoring in a neurosurgical center is essential, and stabilization without further improvement or secondary deterioration should prompt immediate surgical intervention.
